# Within- and between-group regression for improving the robustness of causal claims in cross-sectional analysis

**DOI:** 10.1186/s12940-015-0047-2

**Published:** 2015-07-10

**Authors:** Bernd Genser, Carlos A. Teles, Mauricio L. Barreto, Joachim E. Fischer

**Affiliations:** Mannheim Institute of Public Health, Social and Preventive Medicine, University of Heidelberg, Ludolf-Krehl-Strasse 7-11, Mannheim, 68167 Germany; Instituto de Saúde Coletiva, Federal University of Bahia, Salvador, Brazil; Centro de Pesquisa Gonçalo Muniz, Fundação Oswaldo Cruz (FIOCRUZ), Salvador, Bahia Brazil

**Keywords:** Causal claims, Cross-sectional studies, Multilevel modelling, Ecological fallacy, Ecological inference

## Abstract

**Background:**

A major objective of environmental epidemiology is to elucidate exposure-health outcome associations. To increase the variance of observed exposure concentrations, researchers recruit individuals from different geographic areas. The common analytical approach uses multilevel analysis to estimate individual-level associations adjusted for individual and area covariates. However, in cross-sectional data this approach does not differentiate between residual confounding at the individual level and at the area level. An approach allowing researchers to distinguish between within-group effects and between-group effects would improve the robustness of causal claims.

**Methods:**

We applied an extended multilevel approach to a large cross-sectional study aimed to elucidate the hypothesized link between drinking water pollution from perfluoroctanoic acid (PFOA) and plasma levels of C-reactive protein (CRP) or lymphocyte counts. Using within- and between-group regression of the individual PFOA serum concentrations, we partitioned the total effect into a within- and between-group effect by including the aggregated group average of the individual exposure concentrations as an additional predictor variable.

**Results:**

For both biomarkers, we observed a strong overall association with PFOA blood levels. However, for lymphocyte counts the extended multilevel approach revealed the absence of a between-group effect, suggesting that most of the observed total effect was due to individual level confounding. In contrast, for CRP we found consistent between- and within-group effects, which corroborates the causal claim for the association between PFOA blood levels and CRP.

**Conclusion:**

Between- and within-group regression modelling augments cross-sectional analysis of epidemiological data by supporting the unmasking of non-causal associations arising from hidden confounding at different levels. In the application example presented in this paper, the approach suggested individual confounding as a probable explanation for the first observed association and strengthened the robustness of the causal claim for the second one.

## Background

An important issue in environmental epidemiology is the robustness of causal claims linking exposures to adverse health outcomes. Strong support for causality arises if dose–response relationships between exposures and outcome at the individual level can be demonstrated. However, exposures (e.g. air pollution, persistent chemicals) are often spatially correlated and vary relatively little within single geographic regions. Thus, to obtain sufficient variance of exposure concentrations researchers often recruit individuals from different geographic areas. Compared to measuring exposure directly at the individual level, such assessment of exposure at the area level offers the advantage of lower costs. This approach, referred to as ecological studies or ecological inference, has emerged as an avenue for studying exposure-health associations at the macro-level [1–4]. However, if causal claims are a research objective [5–8], investigators must focus on individual-level exposure-outcome analysis (“biologic inference”) [1]. In most cases individual-level associations cannot be deducted from group-level associations, a phenomenon referred to as ecological fallacy or cross-level bias. Cross-level bias arises from “context effects” (i.e. effects of neighbourhoods on the individual-level exposure-outcome relationships) or from confounding or biases arising differentially at the individual or group level [9–11].

Simple statistical analysis of individual-level data also fails to offer straightforward solutions due to the hierarchical clustering of determinants on different levels, e.g. ecological factors such as environmental exposure concentrations and individual factors such as daily intake, absorption and excretion. To address these issues, researchers increasingly employ hierarchical modelling techniques (multilevel modelling), which allow covariates at different levels [12–14] to be included. However, in cross-sectional data analysis, even these advanced approaches have limitations with regard to the robustness of causal claims. The resulting estimate from such multilevel analysis is a single coefficient for the exposure-outcome relationship that fails to disentangle within-group effects from between-group effects.

The present work describes an approach, which partitions within- and between-group relations. Originally developed in the social sciences, the methodology is *within- and between-group regression* (WBGR) [15]. For example, the average intelligence quotient (IQ) of a school class commonly affects the individual-level association between the IQ and learning performance of students. However, this WBGR approach is rarely applied in environmental epidemiology [16].

The aim of our paper is to generalise the WBGR approach for epidemiological studies where individuals are recruited from different geographical areas and where environmental exposure varies between areas. In these studies, the variation of exposure within and between areas is affected by different factors. Between-area variation results from the effect of area-specific variables (i.e. the magnitude of environmental exposure concentration). Thus, exposure-outcome relations analysed within areas (individual level) and between areas (group level) may yield different results because the relations are prone to different sources of bias or to the presence of a context effect. The latter implies that the average level of a given group affects the individuals’ within-group relation (e.g. the effect of neighbourhoods on individual-level exposure-outcome relations). In the present paper, we illustrate this WBGR approach using a dataset from a cross-sectional study, which primarily investigates whether serum concentrations of the chemical substance perfluorooctanoic acid (PFOA) affected different health-related outcomes.

## Methods

We briefly introduce the basic concepts of WBGR for the applied researcher; mathematical details of the procedure are described in the appendix.

The conventional multilevel approach for clustered data employs equation (1)

1 $$ {Y}_{ij}={\alpha}_{00} + {\beta}_{10\ }{x}_{ij} + {U}_{0j}+{R}_{ij} $$

which incorporates an intercept *α*_00,_ a fixed effect *β*_10_ denoting the total effect observed at the individual level and a random effect of the group *U*_0*j*_; *R*_*ij*_ denotes the residual error term at the individual level within group *j*. Researchers usually include additional covariates at both individual and group levels to minimize the residual error. If the observed total effect *β*_10_ (TE) has exactly the same magnitude as the association observed within areas, the within-group effect (WGE), and as the association observed between area-average values, the between-group effects (BGE), the analysis could safely stop here, as there is little evidence for confounding on the individual or area level. However, equation (1) does not provide this information.

### Within- and between-group regression

The first step of WBGR is to elucidate whether there is homogeneity of the different effect estimators described above, i.e. whether TE = WGE = BGE. We achieve this by introducing an additional term into equation (1), namely, the group average of the individual-level exposure concentrations (equation 2):

2 $$ {Y}_{ij}={\alpha}_{00} + {\beta}_{10\ }{x}_{ij}+{\beta}_{01\ }{\overline{x}}_{.j} + {U}_{0j}+{R}_{ij} $$

In equation (2) the parameter *β*_01_ quantifies the difference between WGE and BGE; the derivation of this relationship is briefly described in the appendix and more mathematical details are described elsewhere [17]. Rejecting the null hypothesis of *β*_01_ = 0 implies an effect of the exposure concentration averaged at the group level beyond the WGE. This test procedure is known in econometrics as the *Hausman test* [18]. For *β*_01_ > 0, the BGE is larger than the WGE, while for *β*_01_ < 0 the BGE is smaller than the WGE (see section below for interpretation).

If *β*_01_ ≠ 0, BGE and WGE should be explicitly estimated by fitting a second model with a slightly different parameterisation. This we achieve by including the deviance of the individual exposure from the group average $\left({x}_{ij}-{\overline{x}}_{.j}\right)$ as a predictor, resulting in equation (3):

3 $$ {Y}_{ij}={\tilde{\alpha}}_{00} + {\tilde{\beta}}_{10}\ \left({x}_{ij}-{\overline{x}}_{.j}\right)+{\tilde{\beta}}_{01\ }{\overline{x}}_{.j} + {U}_{0j}+{R}_{ij} $$

now explicitly estimating *BGE*$\left({\tilde{\beta}}_{01} = {\beta}_{10} + {\beta}_{01}\ \right)$ and *WGE*$\left({\tilde{\beta}}_{10} = {\beta}_{10}\right)$*.*

The extension of WBGR to more complex study designs with more than two levels of clustering is straightforward, as illustrated in the following example. A multi-centre study recruited students from different schools. To disentangle within-class effects, between-class effects and between-school effects, we introduce two additional random effects at the class and at the school level with their respective aggregated exposure levels. Expanding equation (3) allows the size of the between-class and between-school effect to be compared to exposure-outcome associations at the individual level. Mathematical details and applications of three- or higher order multilevel modelling are described elsewhere [17].

#### Adjustment for residual within-group clustering

In multilevel modelling, random effects are the preferred approach to address residual within-group clustering [13, 19]. Conceptually, the random variable represents the effects of all unobserved determinants at the group level. Presence of clustering should be tested by a hypothesis test based on an estimate of the variance of the random effect. Since random-effect modelling has stringent data assumptions (e.g. sufficient number of groups, distributional assumptions of the random effect), robust alternatives such as generalised estimating equations (GEE) or robust variance estimation are often preferable [20–22].

#### Interpretation of WGE and BGE

The simplification above does not account for clustering of individuals within groups, which is examined by determining the intra-class correlation coefficient (ICC). In the classical example of examining the relationship between IQ and academic achievement in students clustered within classes, WGE is the impact of the students’ IQs on the performance score averaged across classes. By contrast, the BGE expresses the effect of the group mean IQ on the group mean performance scores. The overall exposure-outcome relation (TE) estimated by equation (1) is a weighted average of the underlying within- and between-group relations with weights proportional to the (ICC) [17]:

4 $$ \mathrm{T}\mathrm{E} = \mathrm{B}\mathrm{G}\mathrm{E}\ *\ \mathrm{I}\mathrm{C}\mathrm{C} + \mathrm{W}\mathrm{G}\mathrm{E}*\left(1-\mathrm{I}\mathrm{C}\mathrm{C}\right). $$

Equation (4) implies that the TE must always be between BGE and WGE. If clustering is substantial (e.g. ICC > 0.7), the TE will be very close to the BGE. However, if there is only little clustering (e.g. ICC < 0.3), the TE will be close to the WGE. The larger the clustering, the better the BGE represents the TE.

#### WBGR and context effects in environmental epidemiology

**Fig. 1:**
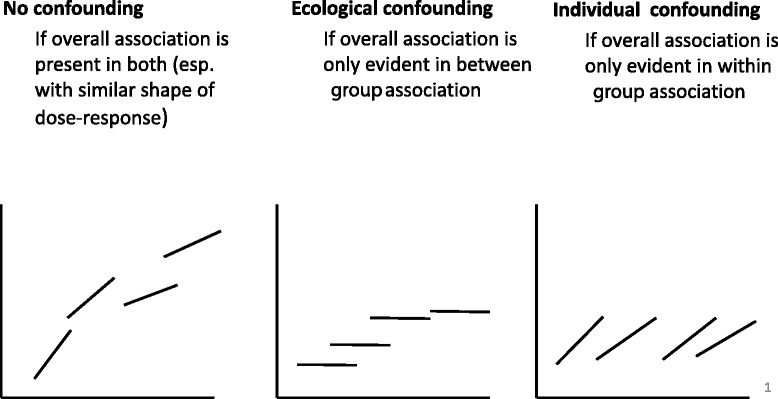
Example patterns of within-and between-group relations. Footnote: The lines are within-group regression lines, x-axis is exposure level, and y-axis is outcome level

True context effects are very common in research on psychosocial determinants of health and have been acknowledged in the social sciences since Émile Durkheim’s seminal work on regional differences in suicide rates more than a century ago [23]. In contrast, in most environmental epidemiological applications, true context effects are rare. A true context effect in the present example of the C8 health project would imply that the biological effect of PFOA on an individual’s health is affected by the average district concentration of PFOA serum exposure beyond the individual PFOA serum concentration. This is rather unlikely, since an individual’s PFOA serum concentration results from the individual’s consumption of polluted water; a person drinking solely bottled water would not have any PFOA exposure at all. Because the effect of the exposure at the macro-level (PFOA concentration in the water district) is reflected in the PFOA exposure in the serum, we can exclude here another causal pathway from the average district level to the health outcomes. Thus, if true context effects are unlikely in terms of biological reasoning but a WBGR analysis reveals the presence of considerable between-group associations (as in our example), at least this part of the total effect cannot be explained by biases or confounding at the individual level. To the extent that we can exclude confounding at the group level, this finding may be interpreted to improve causal claims as illustrated below. In contrast, if the between-group effect estimate is negligible in comparison to the within-group effect or the individual level outcome-exposure association, the presence of bias and non-causality is more likely (Fig. 1) [9].

### An application example – the C8 Health Study

To illustrate the WBGR approach, we analyse data from a large cross-sectional study, the *C8 Health Project*. The study was approved by the *London School of Hygiene and Tropical Medicine Ethics Committee* and is one of the *C8 Science Panel studies;* details of the study design are described elsewhere [24]*.* Briefly, the aim of the study was to elucidate the possible association between the toxic pollutant PFOA and intermediate health outcomes (biomarkers) and clinical outcomes in 69,030 people living in different water districts exposed to environmental pollution by a chemical plant emitting PFOA used in the manufacturing of fluoropolymers. Eligible study participants were recruited between August 2005 and August 2006 in the states of Ohio and West Virginia, USA. Individuals were eligible to participate if they had consumed water for at least one year between 1950 and 2004 while living, working or going to school in one of the six water districts, in an area of private water sources or in areas of documented PFOA pollution (participation rate 80 %). A separate analysis identified residence in one of the contaminated water districts as the strongest predictor of individual PFOA serum concentration [25]. As supporting the causal claim of linking PFOA pollution to health outcomes was a major objective, researchers decided to use the individual’s PFOA serum concentrations as the exposure measure and a variety of health measures as outcomes, including an array of intermediary biomarkers [26–30]. For the present application example we selected a subpopulation of the *C8 Health Project* study population consisting of 25,817 adults (> = 18 years) that were stable residents of six different water districts with different PFOA exposure concentrations in the drinking water.

**Fig. 2:**
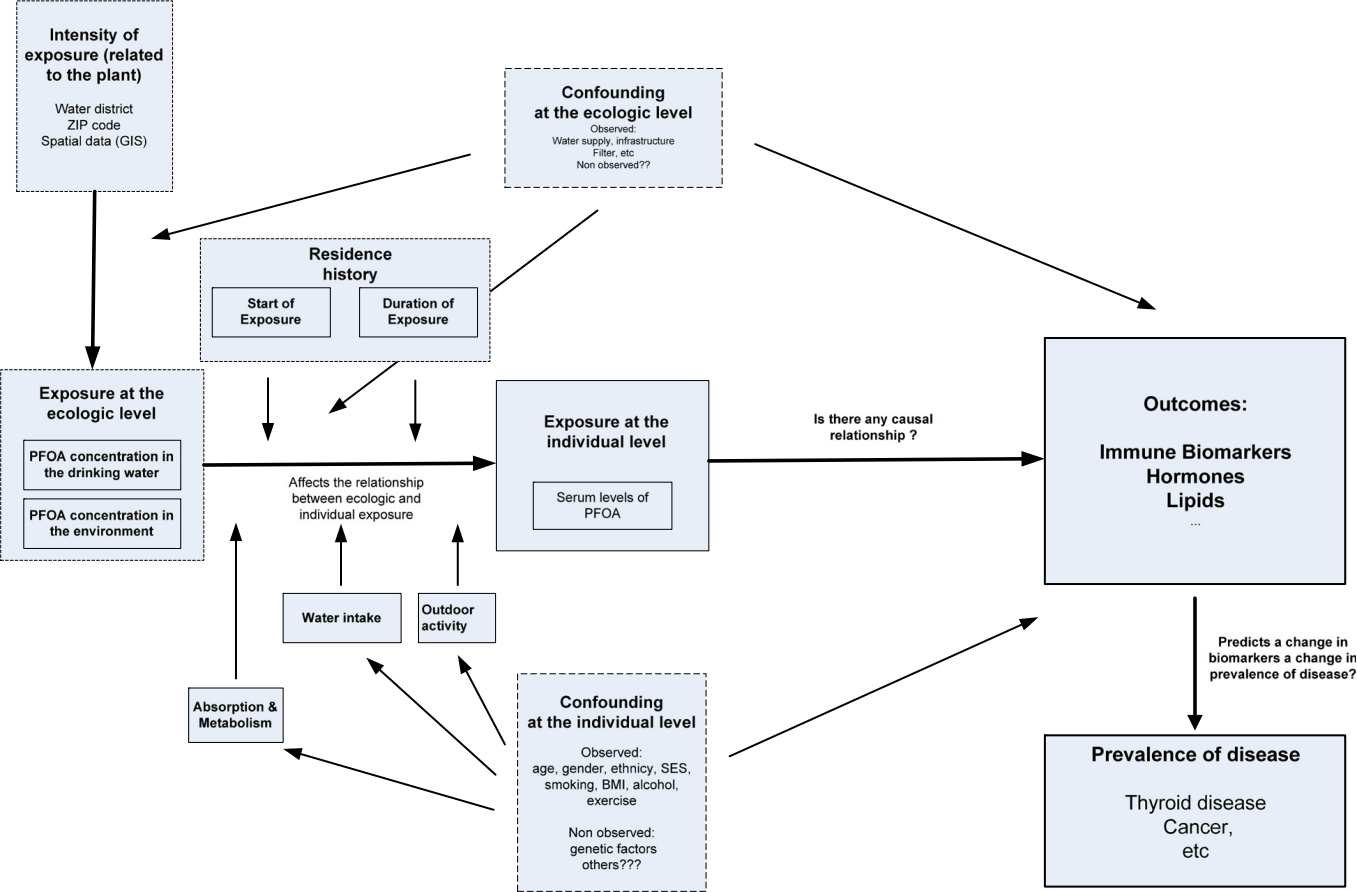
Conceptual model of the C8 Health Project. Footnote: Dotted rectangles indicate larger not directly observable causal constructs

Individual PFOA serum concentrations ensued primarily from water intake from a contaminated drinking water supply. However, an individual’s serum concentrations can ensue both from regional characteristics (i.e. between-group differences in drinking water concentration of PFOA) and from multiple individual factors affecting the bioaccumulation of PFOA (i.e. within-group differences, such as genetic factors, amount of daily water intake, duration of residence in the area). Thus, the exposure was the result of a complex causal chain of exposure determinants acting on different levels (Fig. 2). As the perfect district level exposure measurement (i.e. drinking water concentrations of PFOA at time of water consumption) was not available, the individual PFOA serum concentration reflecting the integrated bioaccumulation over time had to be used as best proxy.

Other research has used such proxies in the concept of instrumental variables [31–33]. An instrumental variable is an operationalisation of an intermediary variable within the causal chain leading from exposure to health outcomes. The key feature of the instrumental variable is its independence of any individual-confounding variable. For example, the association between LDL cholesterol (LDL-C) and cardiovascular disease may be confounded by a myriad of individual behaviour variables, which lead both to elevated LDL-C levels and increased risk of cardiovascular disease. However, genetic variations leading to higher LDL-C levels are very unlikely to be confounded by behavioural variables acquired after birth. Likewise, in the present example, district-average PFOA serum concentrations may be employed as an instrumental variable for PFOA drinking water exposure, as long as individual confounders (e.g. genetic variation in metabolism or individual factors affecting water intake) are randomly distributed across water districts.

#### Applying WBGR to the data of the C8 Health Project

We conducted data analysis in several steps. First, we tested different linear models (total and stratified by district), assuming log-linear and log-log relations. All models were adjusted for potential individual-level confounding variables (age, gender, body mass index, frequency of exercise, alcohol consumption, month of measurement). Secondly, we visualised the relationship pattern by bar plots showing the fitted marginal means of outcomes vs. deciles of PFOA (total, stratified by district and aggregated by district). Third, we tested for heterogeneity of within-district slopes across districts by including interaction terms. Fourth, we assessed whether within- and between-district associations were different by using the WBGR approach, which incorporates the average PFOA level of each district as an additional explanatory variable. If we found heterogeneous BGE and WGE, we fitted a second model using the deviance of individual PFOA exposure from the average PFOA exposure in the district. Finally, we tested for the presence of residual within-district clustering by estimating the variance of the random effect.

For the present illustration of the WBGR concept, we deliberately selected two biomarkers, which showed different patterns of within- and between-district relations: *lymphocyte count*, where we found evidence that the observed association is non-causal, and *C-reactive protein (CRP)*, where we found consistent within- and between-district associations.

## Results

**Fig. 3:**
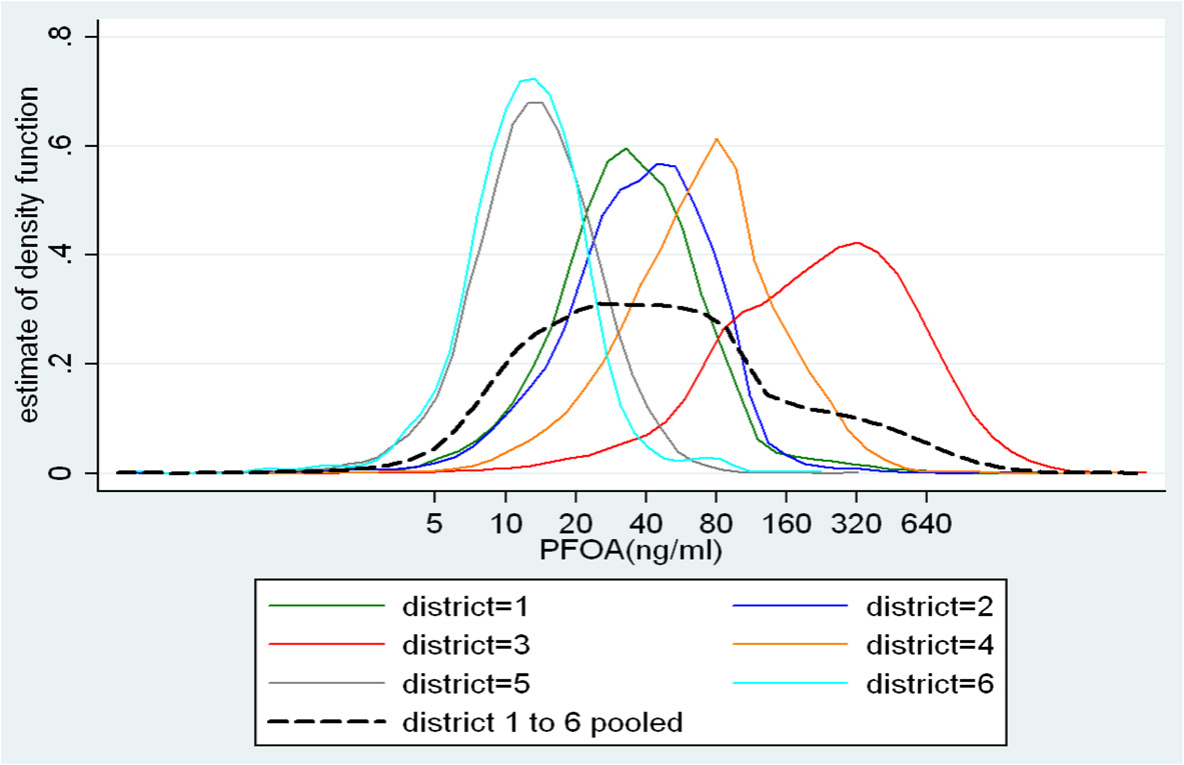
Distribution of PFOA serum concentrations in the water districts

Serum concentrations of PFOA (measured in ng/ml) varied substantially across the six water districts (mean of district means = 89.8, SD = 118.0, min = 15.0, max = 324.2) and among the 25,817 individuals (mean = 102.9, SD = 187.7, min = 0.25, max = 4751.5). As expected, individual PFOA exposure concentrations clustered substantially within water districts (ICC = 46 %, Fig. 3). Likewise, we observed for CRP serum concentrations (measured in mg/l) substantial variance between districts (mean of district medians = 1.7, SD = 0.2, min = 1.5, max = 2.0) and between individuals (median = 2.4, iqr = 2.8, min = 0.2, max = 10.0). By contrast, lymphocyte counts showed very small variation between districts (mean of district means = 2.08, SD = 0.03, min = 2.04, max = 2.12) but strong variation between individuals (mean = 2.07, SD = 0.66, min = 0.20, max = 10.70). Calculation of ICC confirmed this observation: we found significant clustering of CRP concentrations within districts (ICC = 2 %) but absence of within-district clustering of lymphocyte count (ICC = 0 %).

Crude multilevel estimates of the association between PFOA serum concentrations, after adjusting for multiple confounders at the district and individual level, revealed significant overall associations (*p* < 0.001) with the two outcome variables (see total effects shown in Table 1). At this point, it was unclear whether these results were due to residual confounding not detected by a regular multilevel analytical approach.

**Table 1:** Results of within- and between-regression modelling of PFOA on immune biomarkers (the C8-Health project, N = 25 817)

		Between-district effect		Within-district effect		Total effect		Model fit				
Outcome	model	beta^a^	SE^b^	beta^a^	SE^b^	beta^a^	SE^b^	R^2^o^c^	R^2^ ^d^	dR^2^ ^e^	R^2^w^f^	R^2^b^g^
CRP	log-linear	−0.232	0.0525	−0.113	0.0403	−0.157	0.0328	21.0 %	21.1 %	0.1 %	20.7 %	90.4 %
	log-log	−31.021	6.3289	−36.605	7.9959	−33.170	6.3668	21.0 %	21.2 %	0.2 %	20.8 %	92.8 %
Lymphocytes	log-linear	−0.012	0.0166	0.065	0.0127	0.036	0.0097	11.4 %	11.4 %	0.1 %	11.2 %	88.2 %
	log-log	−1.365	1.9997	23.091	2.5265	8.045	3.8012	11.4 %	11.5 %	0.1 %	11.4 %	88.4 %

^a^: regression coefficient of PFOA * 1000

^b^: standard error of regression coefficient * 1000

^c^: coefficient of determination of a model only including covariates without PFOA

^d^: coefficient of determination of a model additionally including PFOA

^e^: difference in R^2^ due to inclusion of PFOA

^f^: coefficient of determination within districts

^g^: coefficient of determination between districts

**Fig. 4:**
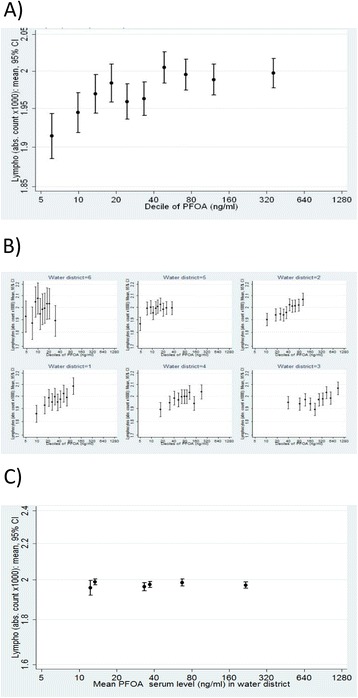
Lymphocyte count per decile of PFOA: **a** Total association observed at the individual level, **b** Within-district associations, stratified by district, **c** Between-district associations (group-level association)

**Fig. 5:**
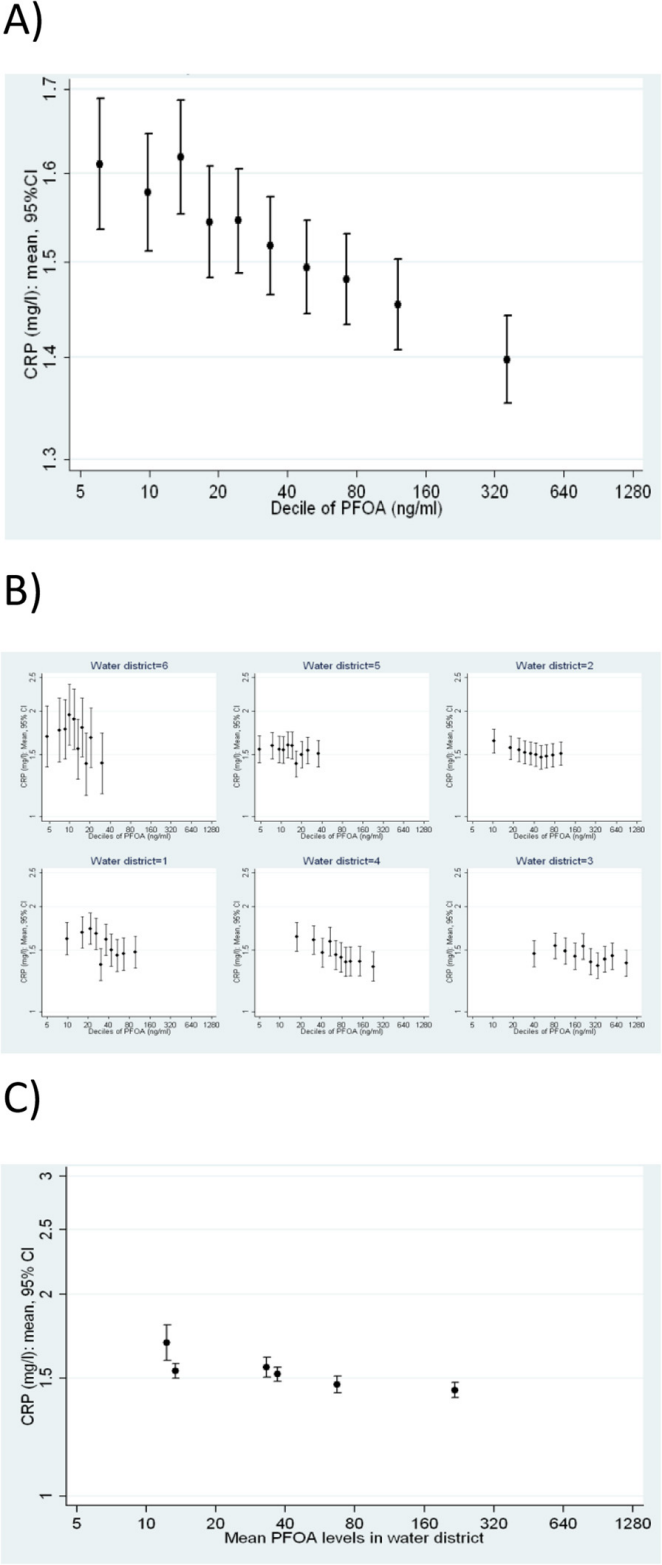
C-reactive protein per decile of PFOA: **a** Total association observed at the individual level, **b** Within-district associations, stratified by district, **c** Between-district associations (group-level association)

Applying the strategy outlined in the methods section by introducing the district-average PFOA serum concentration as the term ${\beta}_{01\ }{\overline{x}}_{.j}$ in equation (2) revealed *β*_01_ as different from 0 for both outcomes (*p* < 0.01). This variance suggested the need for further exploration using the WBGR approach. Estimating the BGE and the WGE using equation (3) revealed a significant BGE for CRP, but no BGE for lymphocyte counts. Results are tabulated in Table 1 and illustrated in Figs. 4 and 5.

In summary, the R^2^ contribution of PFOA was very small and quite similar for log-linear and log scales. For CRP, we observed consistent slopes within all districts (Fig. 5, panel b); slopes were less consistent for lymphocytes within district, showing a saturation effect in some districts (Fig. 4, panel b). If PFOA causes the observed relationship, we would also expect to see an association at the aggregated level (i.e. between districts shown in panel c). This was the case for CRP, where we also observed a clear trend on the aggregated level (Fig. 4c) but not for lymphocyte counts (Fig. 5c). Additionally, we found that the significant clustering of CRP concentrations within districts (ICC = 2 %) disappeared after adjusting for PFOA and other covariates, corroborating the hypothesis that part of the CRP variation is explained by heterogeneous PFOA concentrations between districts. Results from WBGR further corroborated this finding; WGE and BGE were of similar magnitude and statistically significant (Table 1). For lymphocytes, we observed heterogeneous WDE and BDE and heterogeneous WDE within districts, both indicating confounding and/or reverse causality.

## Discussion

We presented WBGR as an approach for statistical analysis of clustered epidemiological data aimed at improving the robustness of causal claims in cross-sectional analysis. We illustrated the application of the approach to data from a large cross-sectional study with strong clustering of individual exposure concentrations (serum concentrations of PFOA) within water districts, which had been contaminated by the emissions from a chemical plant. By disentangling the exposure-outcome relations observed within- and between-groups, such as individuals living in a particular geographical area, the approach may reveal bias in estimates and indicate spurious non-causal exposure-outcome associations. We introduced the basic statistical concepts, discussed the ideas of context effects and cross-level bias, and presented a two-step modelling strategy for practical data analysis within the multilevel framework. From the PFOA study we chose two biomarkers (lymphocyte count and CRP) to illustrate how the approach can be used to improve the robustness of causal claims in cross-sectional analysis; further application examples in the same study are described elsewhere [28–30]. The lymphocyte count showed a strong within-group relation with PFOA but no between-group relation. Thus, we interpreted the observed within-group pattern as a result of individual confounders, i.e. drinking water consumption or absorption/excretion of PFOA (e.g. genetic factors), rather than as due to a causal effect of PFOA exposure. In contrast, for CRP we found consistent and significant within- and between-district associations and thus support a causal claim for the effect of PFOA on CRP. In line with this result, we observed a slight clustering of CRP within districts. This may be interpreted as arising from risk-factor exposure at the group level (e.g. PFOA concentrations in the drinking water assumed to be constant within a particular water district).

The WBGR approach using the district average PFOA serum concentrations as a proxy to estimate the unknown district drinking water concentration at the time of water consumption is related to the statistical framework of instrumental variables [31–33]. An example from the medical literature is the research about the association of CRP and cardiovascular disease. Until genetic Mendelian randomisation studies were performed that introduced genetic factors as instrumental variables to predict biomarkers, it was unclear whether the observed association was causal (i.e. whether CRP causally affects the risk of cardiovascular disease or whether it is merely a marker for a previous cardiovascular). In the present study, district-average PFOA serum concentrations were more proximate to the true exposure (district PFOA water concentration at the time of water consumption) than the individual’s PFOA serum concentration. Another application in epidemiology could be the elucidation of the association between unfavourable psychosocial work characteristics and adverse health outcomes, e.g. the association between work-related perception of stress and cardiovascular disease [34]. If aggregated perceived stress perception levels at the department or company level were available, such aggregated value would be more proximate to the unobservable psychosocial adversity of specific work-settings.

The suggested approach is only applicable if there is some clustering of individual exposure concentrations within water districts or units of aggregation (ICC > > 0 and ICC < < 1). The WBGR approach turns a nuisance in straightforward multilevel regression analysis into an advantage for in-depth analysis supporting or refuting causal claims in cross sectional analysis. In the present study, individual PFOA exposure concentrations were substantially clustered within water districts (ICC = 46 %, Fig. 3), a not surprising finding since the level of environmental pollution with PFOA was different in the water districts. This high intra-district correlation seemed to be a problem because spatial autocorrelation in exposure may correlate with spatial autocorrelation in disease (e.g. due to spatial clustering in health provision, screening take-up or other risk factors). However, disentangling within- and between-district relations by multilevel modelling helped to reveal spurious, non-causal associations (as demonstrated by the example of biomarker lymphocyte counts). Since a true context effect was unlikely in our environmental example, we interpreted heterogeneous between- and within-district relations as an indicator for estimation bias and non-causality of the observed associations. Individual variation in each water district may have resulted from variation in daily tap water intake and other factors affecting the bioaccumulation of PFOA. In contrast, the between-group relations obtained by analysing the data at the district level are robust against these individual confounders.

Further applications of WBGR approach in environmental epidemiology are illustrated in the following hypothetical example. Assuming that simple regression analysis shows an association of PFOA with headaches, an individual-level analysis alone does not clarify whether a) PFOA causes (or prevents) headaches or b) “reverse causality” is present, i.e. people with headaches have a different water intake than healthy individuals and thus have different levels of PFOA. In case (a), we would find an association between PFOA and headaches both within and between areas since the higher average PFOA within a given area would increase the prevalence of headaches in this area. In contrast, in case (b) we would find a relation within each district, but between districts there would be little or no correlation as most of the individual PFOA variation is explained by the PFOA concentration in the water supply. Another reason would be the presence of a third (confounding) factor, which was associated with both PFOA and prevalence of headaches. For example, alcohol use may cause headaches and affect water intake. In that case, a naive individual-level analysis might suggest that PFOA prevented headaches, even if the prevalence of headaches was not lower in a district with lower PFOA exposure.

A major limitation of WBGR is that it improves the robustness of causal claims only indirectly by showing up non-causal associations, which are likely due to bias-neglecting confounders and/or effect modifiers on different levels. Homogeneous between- and within- group relations are a necessary but insufficient condition for assessing causal links. Other criteria are needed to further support the causal claim; however, they can often only be assessed by conducting longitudinal studies, for example, a temporal relationship, plausibility, consistency and strength of association. Further details are described in a systematic approach originally elaborated by Hill [5]. A further limitation to the approach is that the average exposure concentrations of geographical areas are not perfect instrument variables as are genetic factors in a Mendelian randomisation study. However, in our application example, detailed environmental experimental and modelling studies [23] substantiated the claim that the average serum PFOA concentrations in a particular water district may serve as a proxy for its PFOA concentrations in the drinking water.

## Conclusion

Our methodological work shows that WBGR is an elegant technique for the statistical analysis of clustered epidemiological data. The statistical approach proposed in this paper may improve the robustness of causal claims of exposure-outcome associations in cross-sectional analysis by unmasking non-causal associations showing up due to hidden confounding. The approach is especially useful for individual-level analysis in environmental epidemiology in which individuals were recruited from different geographical areas with heterogeneous levels of environmental exposure.

## Appendix

### Mathematical details of within- and between group regression

For a continuous outcome *Y* and an explanatory variable *X* (with observations of individual *i* clustered within groups *j)* we obtain the multilevel regression model

A1 $$ {Y}_{ij}={\alpha}_{00} + {\beta}_{10\ }{x}_{ij} + {U}_{0j}+{R}_{ij}. $$

For simplicity, model (A1) only incorporates one individual-level explanatory variable (*X*); extensions for more explanatory variables at both individual- and group-levels are straightforward. Model (A1) is called a *mixed-effects model,* incorporating an intercept *α*_00 ,_ a *fixed* effect *(β*_10_*)* quantifying the effect of the explanatory variable X and a *random* effect *U*_0*j*_, a random variable which follows a presumed theoretical distribution (usually normal or gamma), and a normal distributed error term *R*_*ij*_. We note that in model (A1) the within- and between group coefficients are forced to be equal, which is an unrealistic assumption for many practical applications. Thus, as an extension, we add the group mean ${\overline{x}}_{.j}$ as an explanatory variable and obtain the more flexible model

A2 $$ {Y}_{ij}={\alpha}_{00} + {\beta}_{10\ }{x}_{ij}+{\beta}_{01\ }{\overline{x}}_{.j} + {U}_{0j}+{R}_{ij}, $$

which allows BGE and WGE to be different.

If we consider model (A2) within a given group, the terms can be reordered as

A3 $$ {Y}_{ij}=\left({\alpha}_{00} + {\beta}_{01\ }{\overline{x}}_{.j} + {U}_{0j}\right)+{\beta}_{10\ }{x}_{ij}+{R}_{ij,} $$

with the group-specific random intercept $\left({\alpha}_{00} + {\beta}_{01\ }{\overline{x}}_{.j} + {U}_{0j}\right)$ and the regression coefficient of X within this group (*β*_10_). The systematic part is the *within-group regression model*

A4 $$ Y=\left({\alpha}_{00} + {\beta}_{01\ }{\overline{x}}_{.j}\right)+{\beta}_{10\ }x $$

By taking the group average on both sides of equation (A3), we obtain the *between-group regression model*

A5 $$ {\overline{Y}}_{.j}={\alpha}_{00} + {\beta}_{10\ }{\overline{x}}_{.j}+{\beta}_{01\ }{\overline{x}}_{.j} + {U}_{0j}+{R}_{ij}={\alpha}_{00} + \left({\beta}_{10} + {\beta}_{01\ }\right){\overline{x}}_{.j} + {U}_{0j}+{R}_{ij}. $$

Model (A5) shows that using this parameterization the term (*β*_10_ + *β*_01_) estimates the BGE. If we reject the null hypothesis (*β*_01_ = 0) we fit a second model (7) incorporating group-centered explanatory variables*,* i.e. variables with mean 0 that measure the individuals’ deviation within a group from the group mean:

A6 $$ {Y}_{ij}={\tilde{\alpha}}_{00} + {\tilde{\beta}}_{10}\ \left({x}_{ij}-{\overline{x}}_{.j}\right)+{\tilde{\beta}}_{01\ }{\overline{x}}_{.j} + {U}_{0j}+{R}_{ij}\ . $$

Model (A6) now explicitly estimates *BGE*$\left({\tilde{\beta}}_{01} = {\beta}_{10} + {\beta}_{01}\right)$ and *WGE*$\left({\tilde{\beta}}_{10} = {\beta}_{10}\right)$.
